# Supplementary material for: Extracellular mRNA transported to the nucleus exerts translation-independent function
Source: Nat Commun. 2021 Jun 16;12:3655. doi: 10.1038/s41467-021-23969-1 (PMC8208975; doi:10.1038/s41467-021-23969-1)
Supplement: Supplementary file 1 — Supplementary Information [file 41467_2021_23969_MOESM1_ESM.pdf]

Supplementary Figure 1

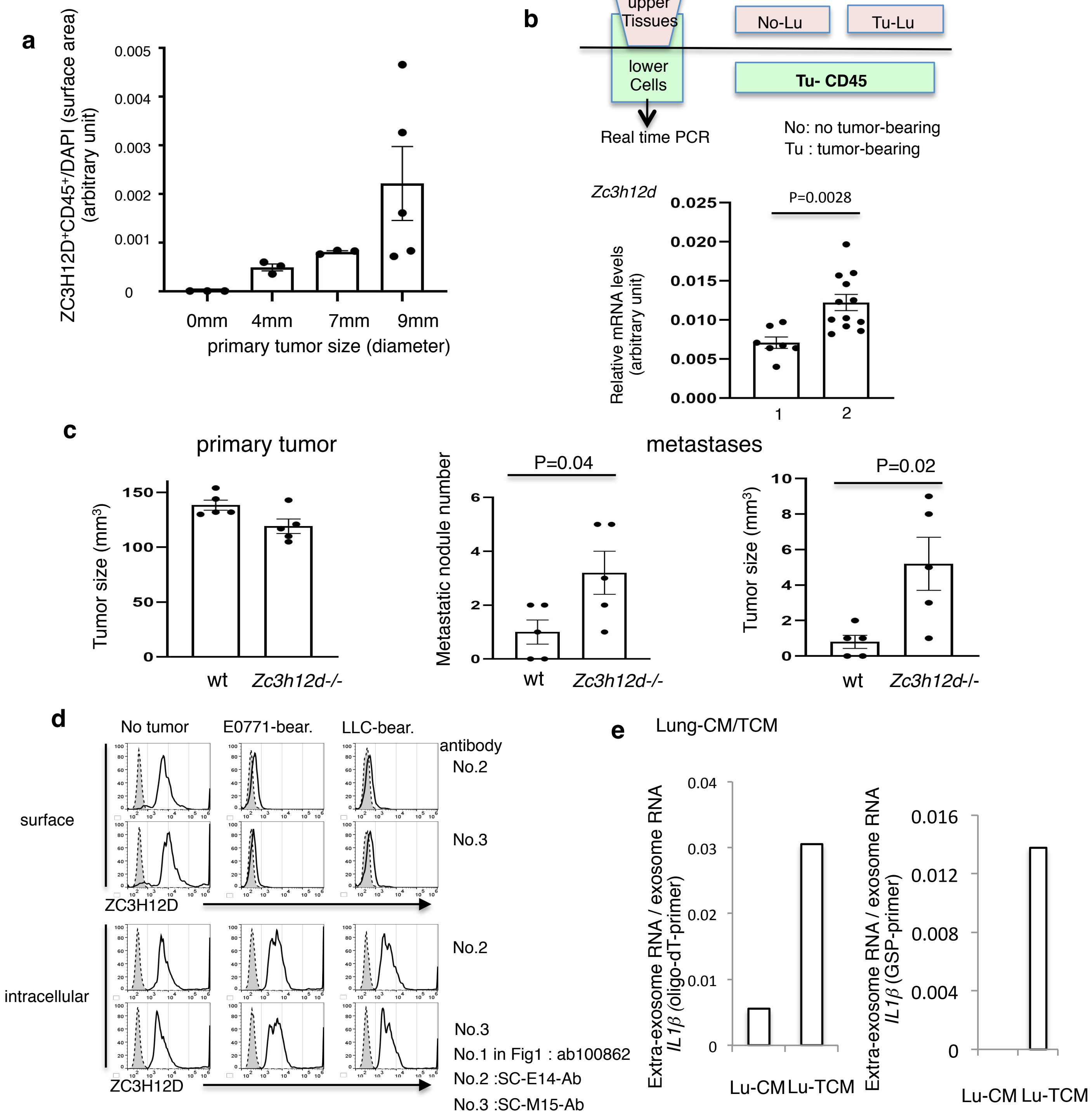

(a) ZC3H12D⁺ leukocytes increased in E0771-bearing mouse lungs during primary tumor growth. IHC examination was conducted on lung tissue taken from mice with primary tumors of the following sizes: 0 mm (n = 3), 4 mm (n = 3), 7 mm (n = 3), and 9 mm (n = 5). **(b)** An assay system used to examine mRNA levels in CD45⁺ leukocytes in bottom wells cultured with lung tissues in top wells (top). *Zc3h12d* expression in leukocytes was stimulated by tumor-bearing lungs. The lungs were obtained from mice without tumors (n = 7) and tumor-bearing mice (n = 12; bottom). (**c**) Primary E0771 tumor growth of wild-type (n = 5) and *Zc3h12d-/-* (n = 5) mice (left). The metastatic nodule number (middle) and total tumor size (right) in each mouse type are presented. **(d)** FACS analysis of surface and intracellular ZC3H12D protein in PBMCs derived from mice without tumors, E0771-bearing mice, and LLC-bearing mice. Three anti-ZC3H12D antibodies were used. **(e)** qPCR analysis of m*IL1β*-mRNA. Ratio of extraexosome RNA compared to exosome RNA in mouse lung culture medium with control CM or TCM. RT was carried out using oligo(dT) (left) and GSP (right) primers. In the graphs, the averages ± SEM, and results of a Student's t-tests (two-sided) or one-way ANOVA with Bonferroni correction are shown. The P values are shown in the figure. Source data are provided as a Source Data File.

Supplementary Figure 2

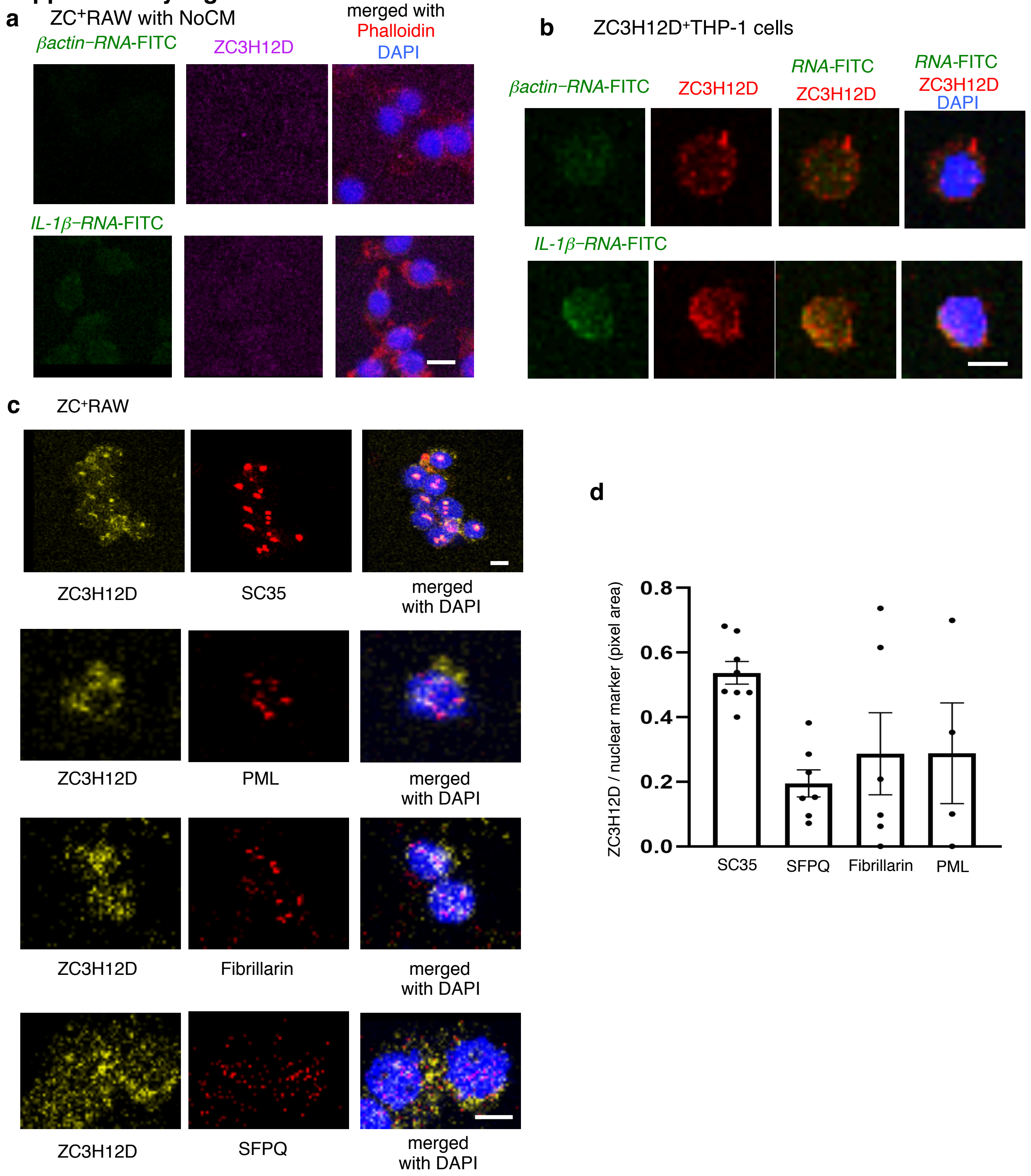

(a) Representative images showing that ZC3H12D on the surface of ZC+RAW was not induced by NoCM. **(b)** Colocalization of *IL1β*-mRNA-FITC and ZC3H12D on ZC3H12D+THP1 cells. **(c)** IHC analysis of colocalization of ZC3H12D and a speckle marker, SC35, in the nucleus of *IL1β*-mRNA-stimulated ZC+RAW cells. In comparison to SC35, IHC analysis reveals that the ZC3H12D signal was less colocalized with another nuclear body marker (PML, fibrillarin, and SFPQ) in the nucleus of *IL1β*-mRNA-stimulated ZC+RAW cells. Anti-PML, fibrillarin, and SFPQ antibodies are for PML body (PML), nucleolus, and paraspeckle, respectively. **(d)** Quantitative analysis of colocalized signals of ZC3H12D and nuclear body markers. (n = 8, 8, 7 and 4 wells for SC35, SFPQ, Fibrillarin and PML, respectively). Bars, 5 μm. In the graphs, the averages ± SEM, and the results of one-way ANOVA with Bonferroni correction are shown. Source data are provided as a Source Data File.

Supplementary Figure 3

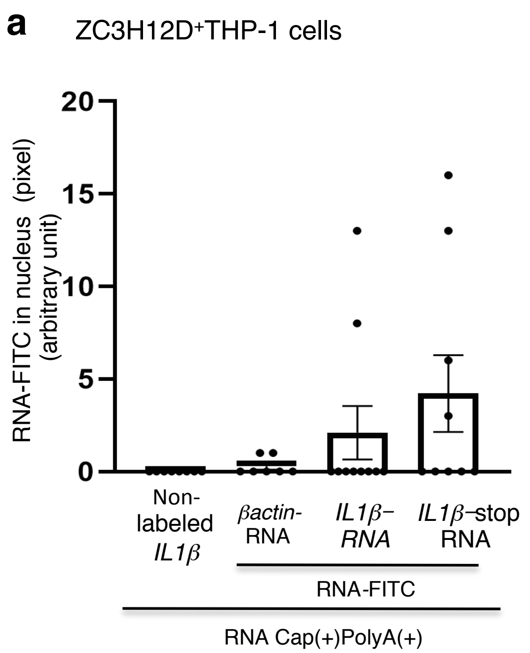

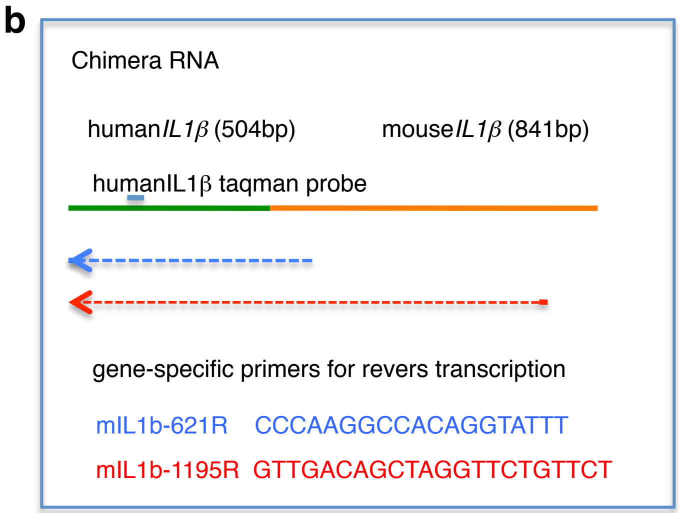

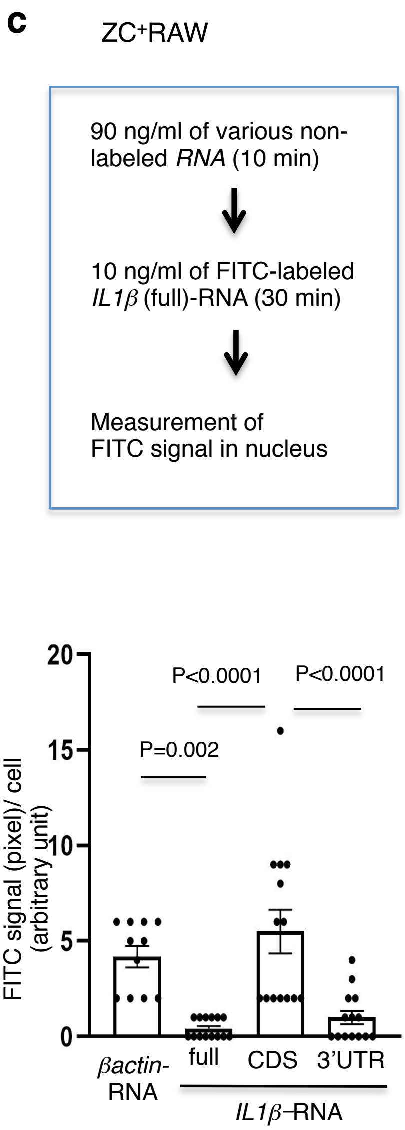

(a) Quantitative analysis of RNA-FITC in the nucleus of ZC3H12D+THP1 cells after application of 10 ng/mL nonlabeled *IL1β*-mRNA, FITC-labeled *βactin*-mRNA, *IL1β*-mRNA, and *IL1β*-stop-mRNA with CP (n = 8, 7, 10 and 9 cells for non-labeled *IL1β*-RNA, FITC-labeled- *βactin*-RNA, *IL1β*-RNA and *IL1β*-stop-RNA, respectively. Each cell image is composed of 15-stacked 3D images). **(b)** Biochemical detection system for *RNA* uptake in the nucleus of mouse ZC+RAW cells using human-mouse-chimera *IL1β*-mRNA*.* Purified RNA from nuclear and cytoplasmic fractions were used as templates for reverse transcription with mGSPs. cDNAs were subjected to real-time PCR analysis using an h*IL1β*-specific TaqMan probe as shown above. Note that this probe does not detect endogenous m*IL1β*. **(c)** Competition assay for *IL1β*-mRNA uptake in the nucleus of ZC+RAW cells. The assay scheme is presented on top. The uptake of *IL1β-full* was inhibited by pretreatment with the 3′-UTR (bottom). Avarages (n = 11, 14, 14 and 14 cells for *βactin*-RNA, full length, CDS, and 3'UTR of *IL1β*-RNA, respectively. Each cell image is composed of 15-stacked 3D images) are shown. In the graphs, the averages ± SEM, and the results of one-way ANOVA with Bonferroni correction are shown. The P values are shown in the figure. Source data are provided as a Source Data File.

Supplementary Figure 4

a

EMSA Probe 1	m*IL1β* (1000-1050)	CGGCCAAGACAGGUCGCUCAGGGUCACAAGAAACCAUGGCACAUUCUGUU
EMSA Probe 2	m*IL1β* (1025-1075)	ACAAGAAACCAUGGCACAUUCUGUUCAAAGAGAGCCUGUGUUUUCCUCCU
EMSA Probe 3	m*IL1β* (1050-1100)	CAAAGAGAGCCUGUGUUUUCCUCCUUGCCUCUGAUGGGCAACCACUUACC
EMSA Probe 4	m*IL1β* (1075-1125)	UGCCUCUGAUGGGCAACCACUUACCUAUUUAUUUAUGUAUUUAUUGAUUG
EMSA Probe 5	m*IL1β* (1100-1150)	UAUUUAUUUAUGUAUUUAUUGAUUGGUUGAUCUAUUUAAGUUGAUUCAAG
EMSA Probe 6	m*IL1β* (1125-1175)	GUUGAUCUAUUUAAGUUGAUUCAAGGGGACAUUAGGCAGCACUCUCUAGA
EMSA Probe 7	m*IL1β* (1150-1200)	GGGACAUUAGGCAGCACUCUCUAGAACAGAACCUAGCUGUCAACGUGUGG
EMSA Probe 8	m*IL1β* (1175-1225)	ACAGAACCUAGCUGUCAACGUGUGGGGGAUGAAUUGGUCAUAGCCCGCAC
EMSA Probe 9	m*IL1β* (1200-1250)	GGGAUGAAUUGGUCAUAGCCCGCACUGAGGUCUUUCAUUGAAGCUGAGAA

b

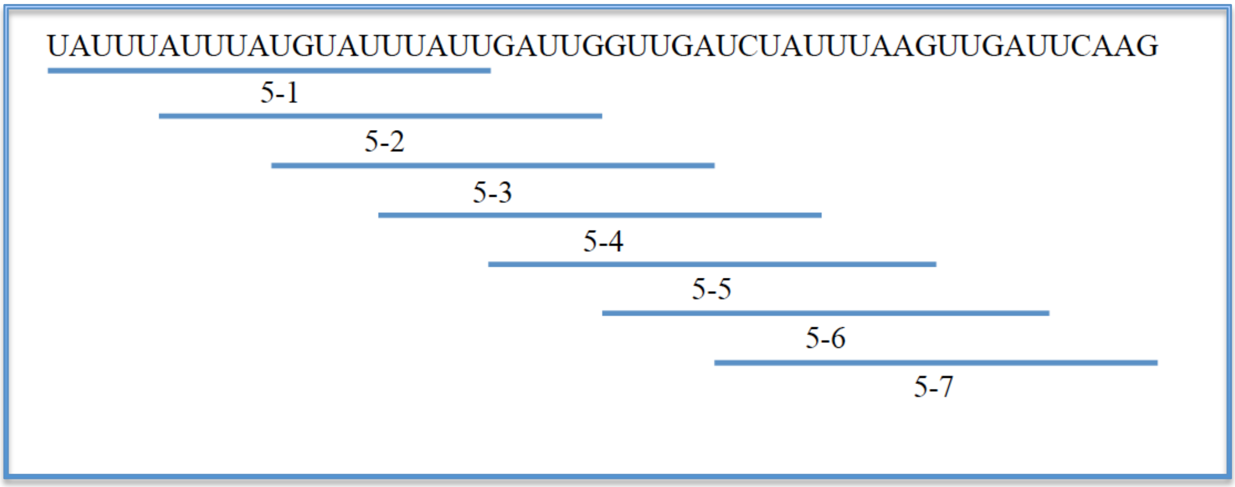

(a) EMSA probes are described in this table. Each number shows the base position in the canonical m*IL1β* transcript.
(b) Nucleotide sequence of EMSA probe 5 and short competitors (5-1–5-7).

Supplementary Figure 5

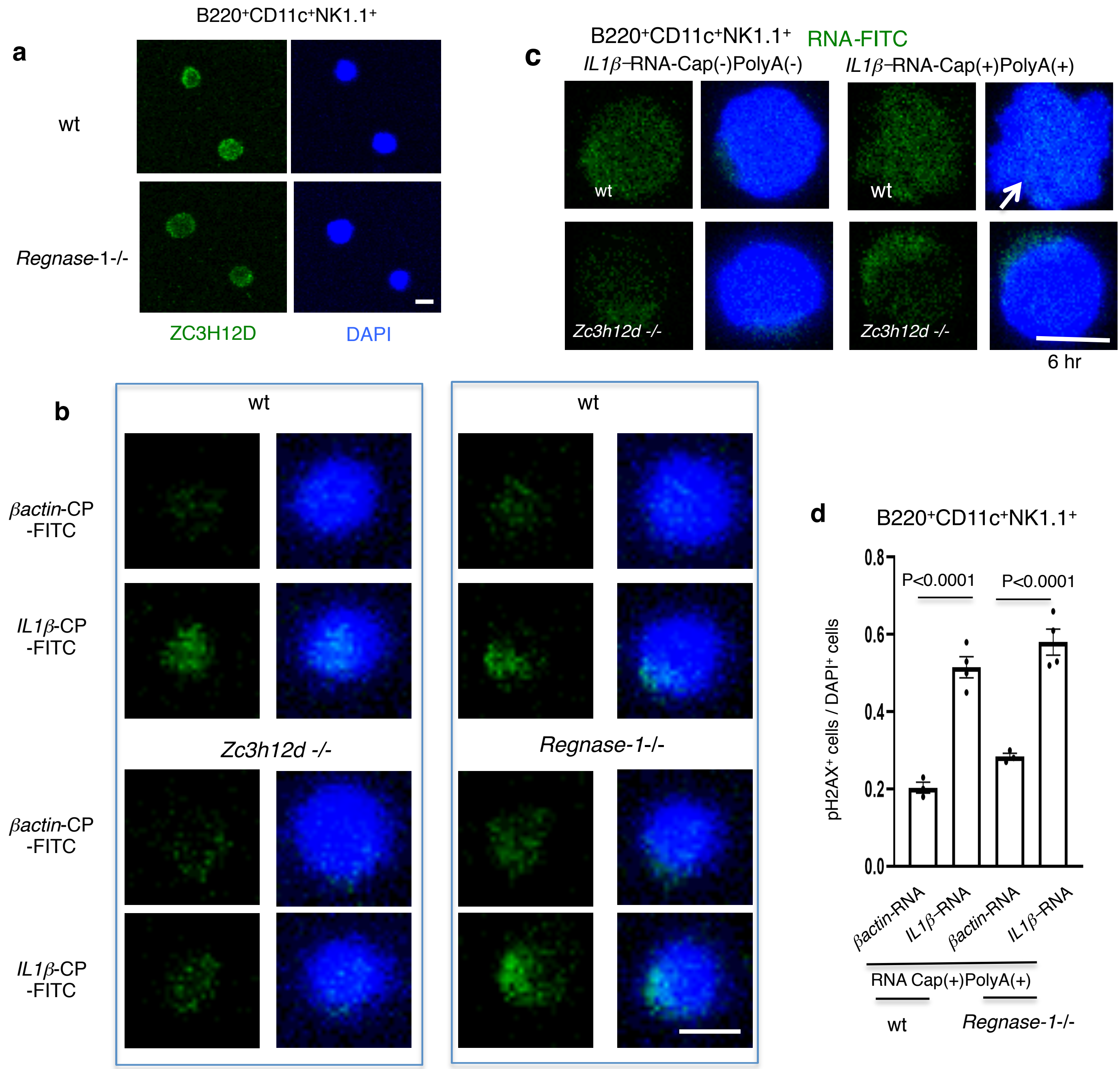

(a) Representative ZC3H12D expression in B220+CD11c+NK1.1+NK cells derived from wild-type and *Regnase-1-/-* (*Zc3h12a*$^{-/-}$) spleens. 3D image analysis using confocal microscopy (Z-stack: 15 images) is presented. **(b)** Signal of RNA-FITC shown in B220+CD11c+NK1.1+ cells derived from wild-type, *Zc3h12d*-/-, and *Regnase-1-/-* mice 3 h after application of 10 ng/mL *βactin*-mRNA-CP-FITC and *IL1β*-mRNA-CP-FITC. The wild littermate was used for each knockout mouse. 3D image analysis using confocal microscopy (Z-stack: 15 images) is presented. **(c)** Representative photo showing the irregular shape of the nucleus 6 h after the uptake of *IL1β*-RNA-CP in B220+CD11c+NK1.1+ cells derived from wild-type mice (arrows). Experiments were repeated twice with similar results. **(d)** Quantitative IHC analysis for phospho-H2AX in the nucleus of B220+CD11c+NK1.1+ cells from wild-type and *Regnase-1-/-* 3 h after application of *βactin*-mRNA and *IL1β*-mRNA (n = 3 and 4 wells for *βactin*-mRNA and *IL1β*-mRNA, respectively). Bar, 5 µm. In the graphs, the averages ± SEM, and the results of one-way ANOVA with Bonferroni correction are shown. The P values are shown in the figure. Source data are provided as a Source Data File.

Supplementary Figure 6

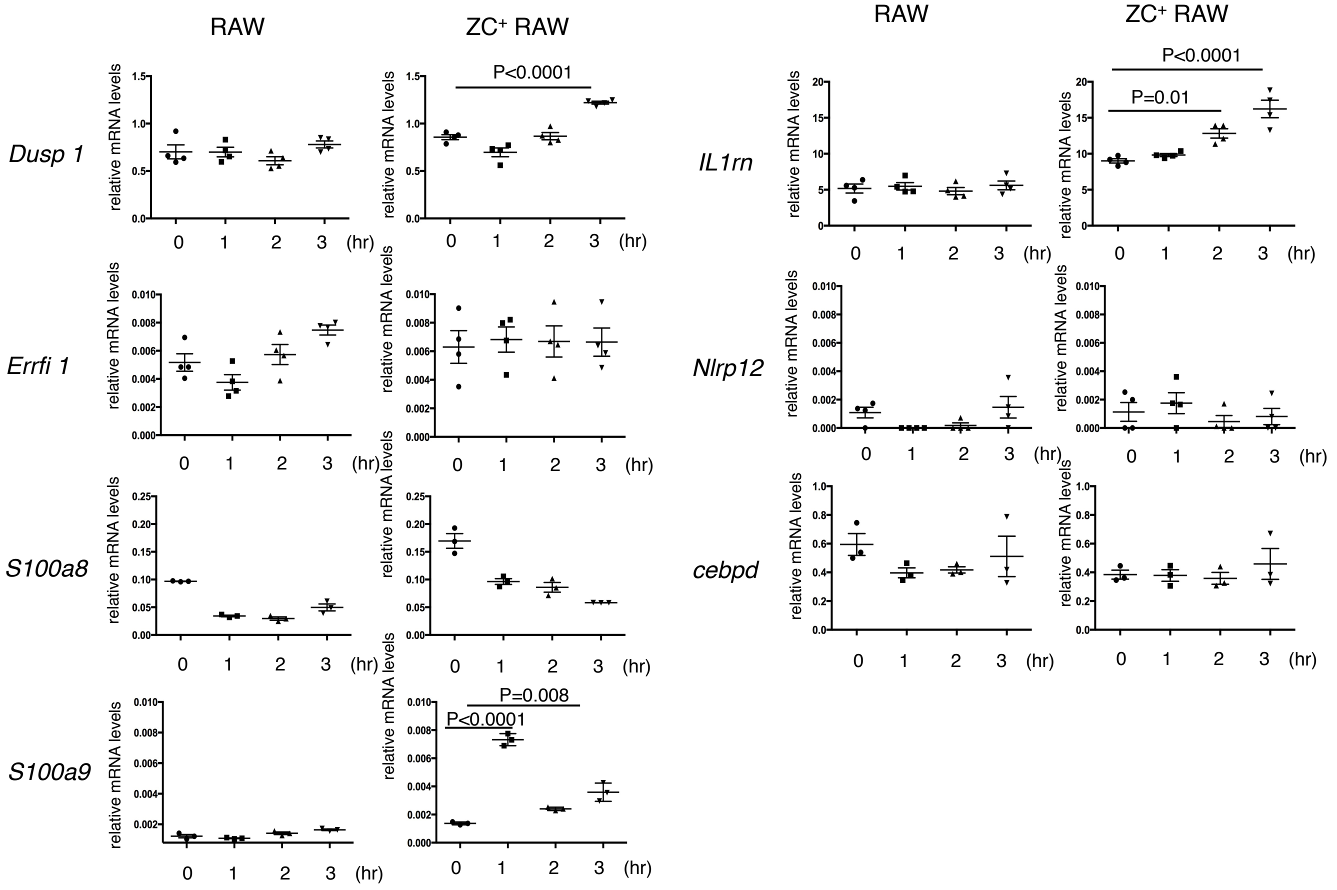

Time course of mRNA expression after treatment with *IL1β*-RNA*.* Vertical axes indicate the relative mRNA levels normalized by *βactin* (n = 4 biologically independent samples for *Dusp1*, *IL1rn*, *Errfi1* and *Nlrp12* and n = 3 biologically independent samples for *S100a8*, *S100a9* and *cebpd*). In the graphs, averages ± SEM and the results of one-way ANOVA with Bonferroni correction are shown. The P values are shown in the figure. Source data are provided as a Source Data File.

Supplementary Figure 7

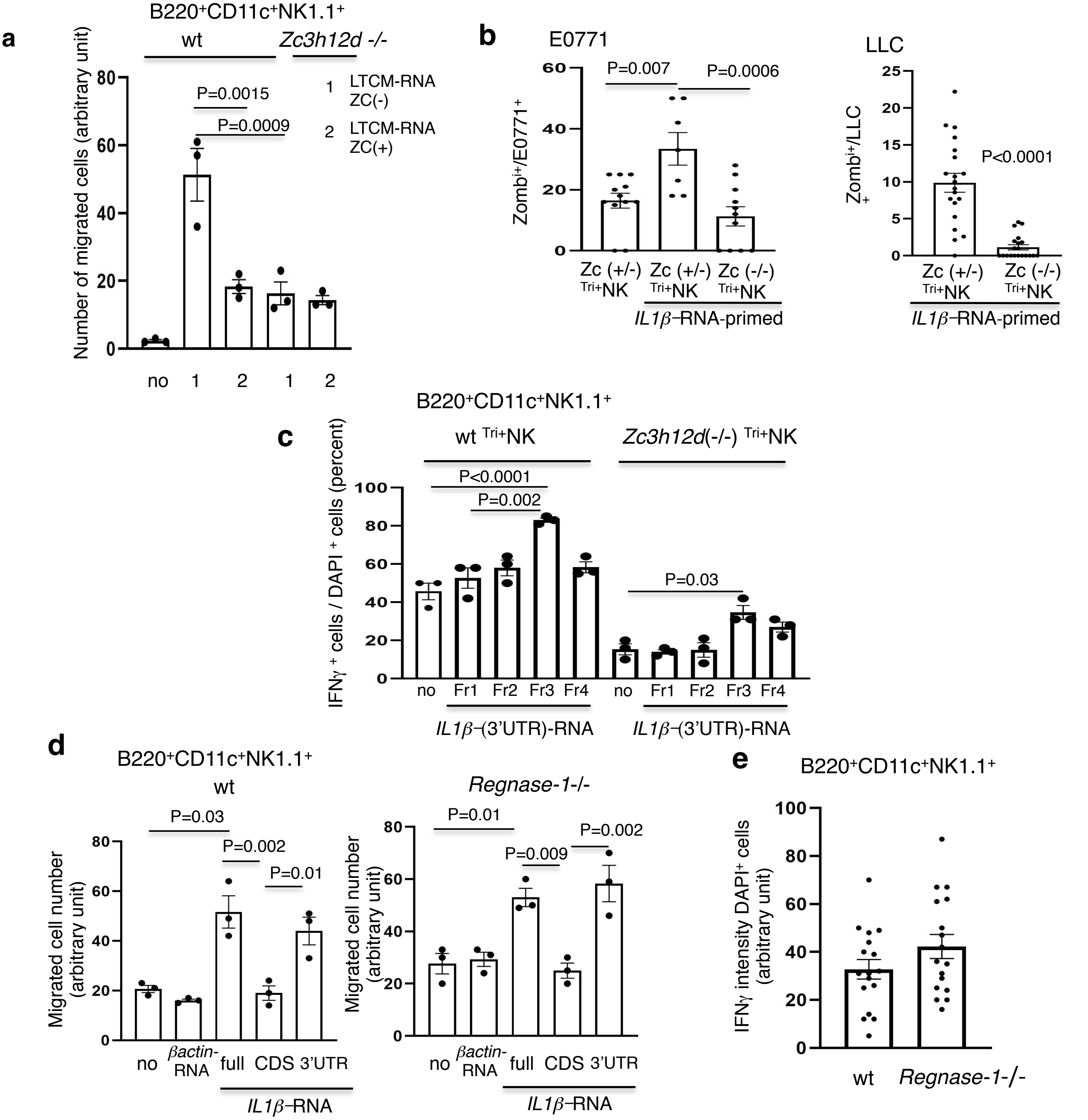

(a) Migration assay for RNA derived from TCM-depleted ZC3H12D-binding RNA. RNA samples were obtained from LTCM passed through a no protein column (No. 1) and a ZC3H12D protein column (No. 2). No, zero RNA. The number of migrated B220$^+$CD11c$^+$NK1.1$^+$ cells of wild and *Zc3h12d-/-* mice are shown (n = 3 wells per group). **(b)** Tumoricidal assay *in vitro* after coincubation of tumor cells with *IL1β*-mRNA-primed B220$^+$CD11c$^+$NK1.1$^+$ cells. *Zc3h12d+/-* and *Zc3h12d-/-* mice were same littermates. B220$^+$CD11c$^+$NK1.1$^+$ is shown as ^{Tri}NK. (left, n = 12, 7 and 11 wells for Zc(+/-), *IL1β*-RNA-primed Zc(+/-) and Zc(-/-), respectively. right, n = 20 wells). **(c)** Upregulation of IFN-γ in B220$^+$CD11c$^+$NK1.1$^+$ cells from TCM-stimulated wild-type or *Zc3h12d-/-* mice with four fragments of 3′-UTR (n = 3 culture dishes per group). **(d)** Migration assay for *IL1β*-mRNA domains and *βactin*-mRNA using wild type and *Zc3h12a-/-* (*Regnase-1-/-*) mice B220$^+$CD11c$^+$NK1.1$^+$ cells (10 ng/mL; n = 3 wells per group). **(e)** IFN-γ in B220$^+$CD11c$^+$NK1.1$^+$ cells from wild-type or *Regnase-1-/-* mice with *IL1β*-mRNA (n=17 cells from 2 wild-type and 3 *Regnase-1-/-* mice). In the graphs, averages ± SEM and the results of a Student's t-test or one-way ANOVA with Bonferroni correction are shown. The P values are shown in the figure. Source data are provided as a Source Data File.

Supplementary Figure 8

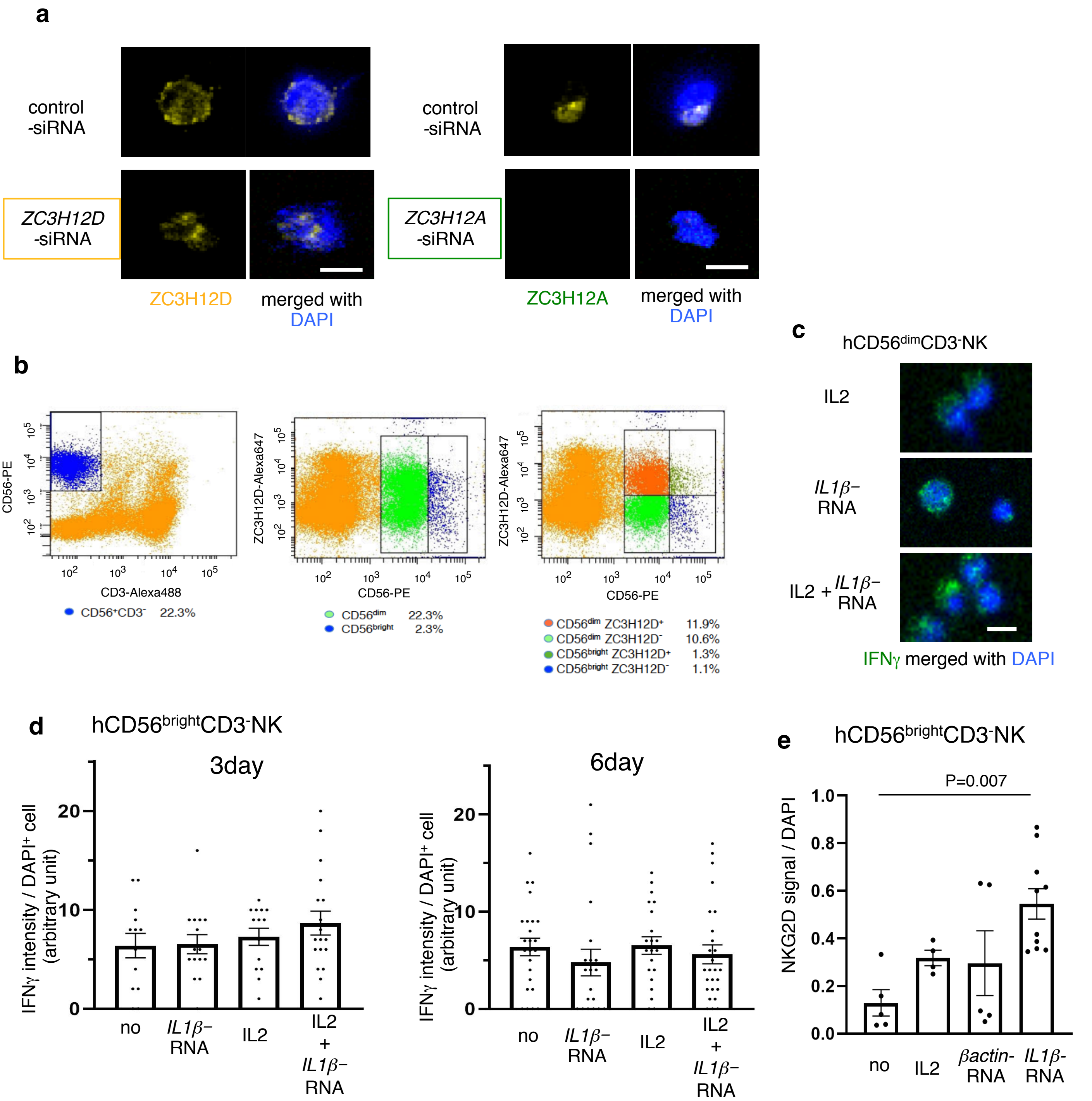

(a) Knockdown of ZC3H12D and ZC3H12A proteins using siRNA in human NK cells. Representative 3D images with IHC staining are presented. Images show the reduction of ZC3H12D and ZC3H12A expression in $CD56^{+}CD3^{-}$NK cells 24 h after electroporation of siRNA. Bar, 5 µm. Experiment was repeated once with similar result. **(b)** FACS analysis of surface ZC3H12D protein in $CD56^{bright}$ and $CD56^{dim}$ NK cells derived from hPBMCs. Gating strategy using anti-ZC3H12D antibody was shown in Supplementary Figure 10. **(c)** Representative photo of IFN-γ staining 6 days after treatment with h*IL1β*-mRNA and IL2 protein for MDAMB231-TCM-pretreated $CD56^{dim}CD3^{-}$NK cells from PBMC. Ten stacked confocal images are shown. Bar, 5 µm. **(d)** IHC analysis of IFN-γ production 3 and 6 days after treatment with h*IL1β*-mRNA and IL2 protein for MDAMB231-TCM-pretreated $CD56^{bright}CD3^{-}$NK cells (bottom left, n = 13, 15, 14 and 18 cells for no, *IL1β*-RNA, and IL2 protein, and *IL1β*-RNA plus IL2 protein, respectively. bottom right, n = 34, 21, 21 and 26 cells for no, *IL1β*-RNA, and IL2 protein, and *IL1β*-RNA plus IL2 protein, respectively). **(e)** NKG2D expression in $CD56^{bright}CD3^{-}$NK cells 6 days after incubation with RNA (n = 5, 4, 5 and 10 wells for no, IL2 protein, *βactin*-RNA and *IL1β*-RNA, respectively). In the graphs, averages ± SEM and the results of one-way ANOVA with Bonferroni correction are shown. The P values are shown in the figure. Source data are provided as a Source Data File.

Supplementary Figure 9 - Repeated Data

Figure 1d

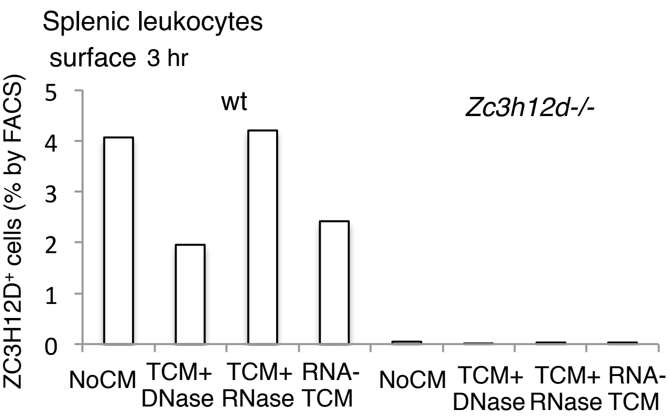

Figure 1h

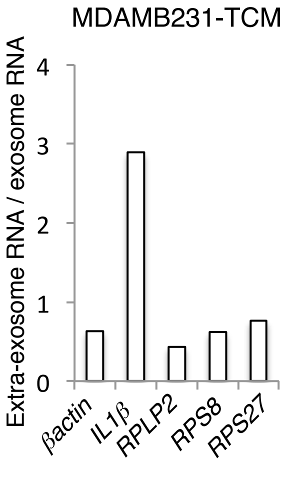

Figure 1i

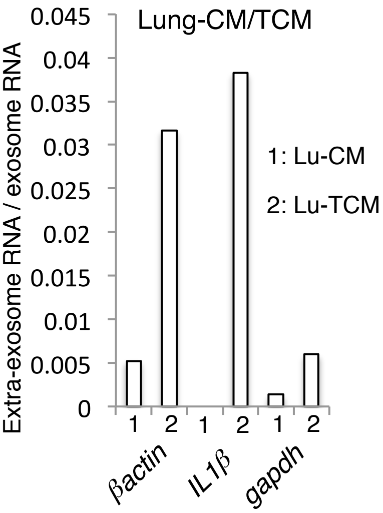

Figure 1j

Tumor-bearing mouse serum

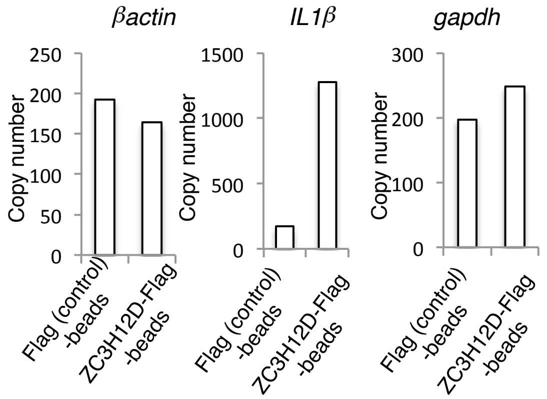

Figure 2b

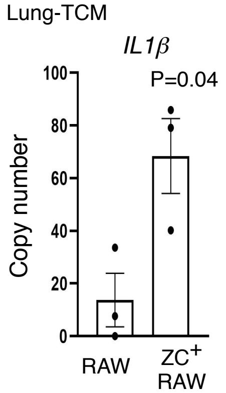

Figure 3a

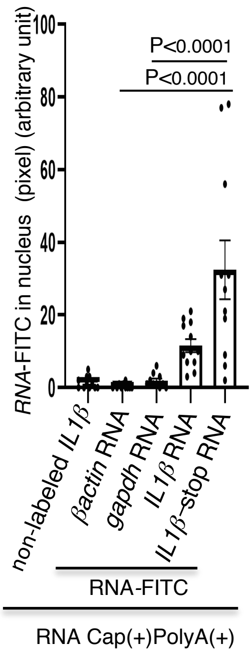

Figure 5f

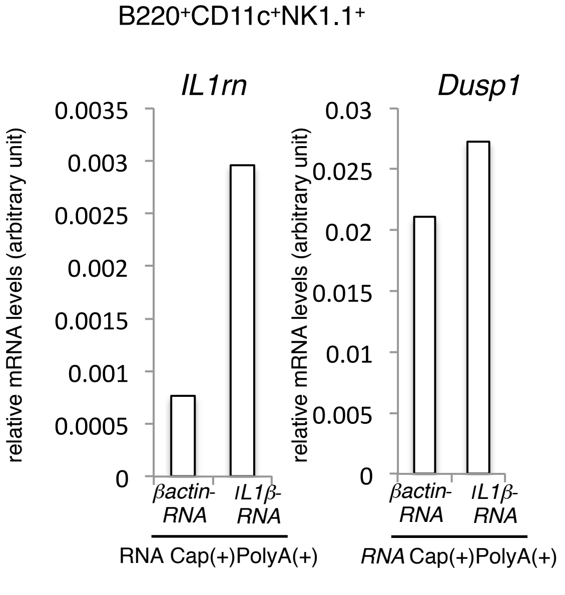

Repeated experiment data for Figures 1d and h to j, 2b, 3a, and 5f. These data show that the key points (*IL-1β* in Figure 1i and j and 2b) were reproduced. (n = 3 biologically independent samples in Figure 2b-repeated data; n=11, 10, 10, 12 and 11 cells for nonlabeled *IL1β*-RNA, FITC-labeled -*βactin*-RNA, *gapdh*-RNA, *IL1β*-RNA and *IL1β*-stop-RNA, respectively in Figure 3a-repeated data) In the graphs, averages ± SEM and the results of a Student's t-test or one-way ANOVA with Bonferroni correction are shown. The P values are shown in the figure.

Supplementary Figure 10 -Gating strategy and validation of anti-ZC3H12D antibody

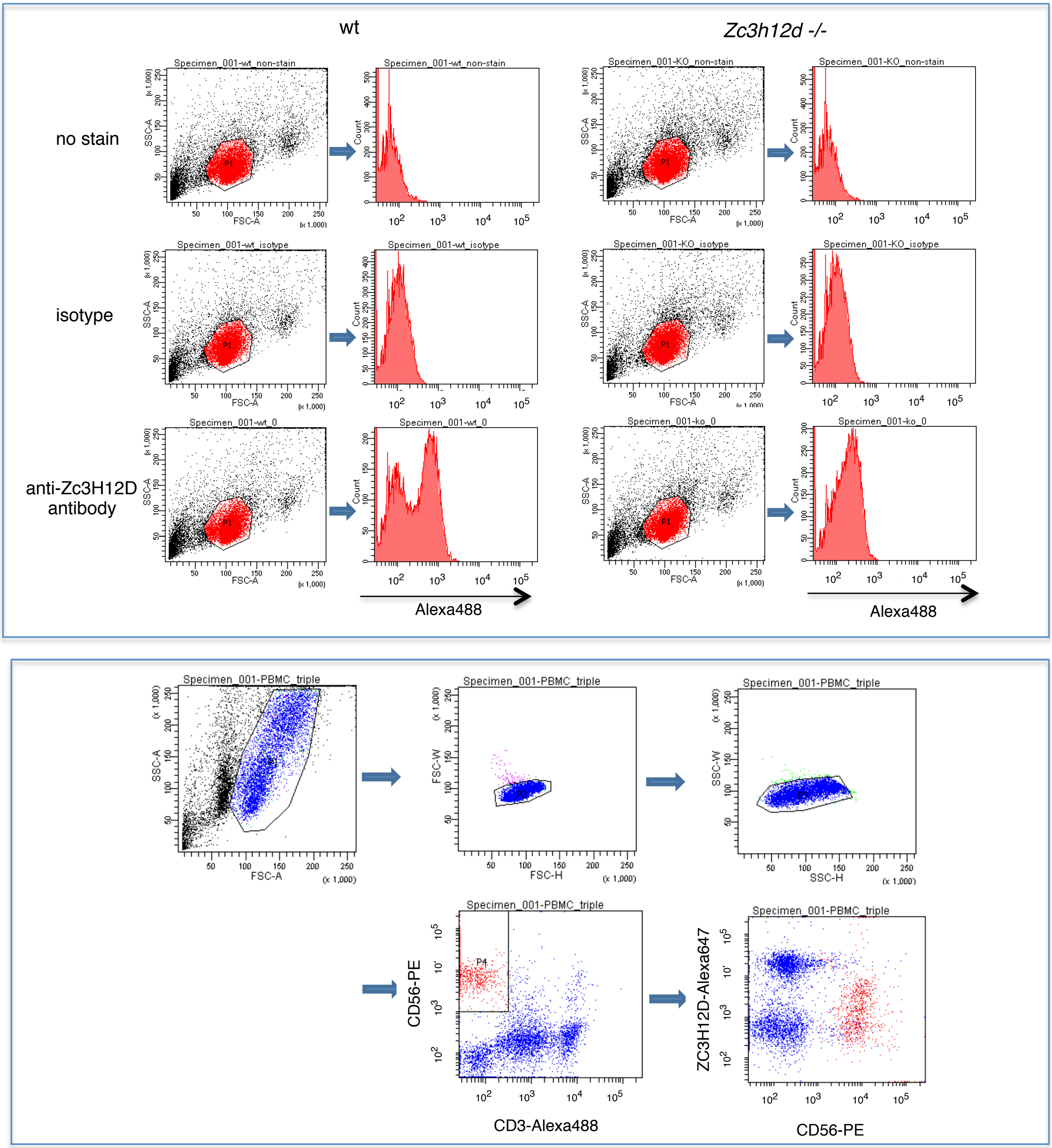

ZC3H12D antibody validation and gating strategy for the mouse samples related to Figure 1a and 1d (top panel). Wild-type mouse spleen cells were analyzed by flow cytometry: (top) no primary antibody, (middle) isotype control, and (bottom) anti-ZC3H12D are shown in left. ZC3H12D knockout mouse spleen cells were also analyzed: (top) no primary antibody, (middle) isotype control, and (bottom) anti-ZC3H12D are shown in right. Anti-Rabbit-IgG-Alexa 488 was used as secondary antibody in the analyses. The gating strategy for the human PBMC cells related to Figure 7a and Supplementary Figure 8 (bottom panel).

SupplementaryTable 1

	lung > liver
Gene symbol	Fold change
Zc3h12a	-0.42
Zc3h12c	-1.87
Zc3h12d	6.19

Gene expression of ZC3H12 family in relocated B220^{+}CD11c^{+}NK1.1^{+}NK cells derived from TCM-stimulated lungs and livers. *Zc3h12d* showed the highest upregulation in B220^{+}CD11c^{+}NK1.1^{+}NK cells that migrated from the liver to the lung compared to those that stayed in the liver in TCM-stimulating mice. (Microarray data were deposited in GSE76235.) As shown in Table S3, *Zc3h12b* might be low. To perform this microarray analysis, cDNAs obtained from five mice were combined before the hybridization process. The fold changes of mRNA levels in lung versus liver were shown.

SupplementaryTable 2

Gene accession	Gene symbol	gene expression value
NM_007393	Actb	999949
NM_001033261	Zfc3h1	999949
NM_011664	Ubb	684039
ENSMUST00000082392	ND1	528615
NM_011359	Sftpc	387841
NM_008361	Il1b	377199
NM_013647	Rps16	239062
NM_001252218	Rpl31	60126
NM_008503	Rps2	51180
NM_011296	Rps18	49266
NM_007621	Cbr2	38421
NM_010106	Eef1a1	37956
NM_029751	Rpl18a	33092
NM_012053	Rpl8	21974
NM_011681	Scgb1a1	19695
NM_011295	Rps12	15829
NM_016959	Rps3a1	15709
NM_011029	Rpsa	15285
NM_009975	Csnk2b	14793
NM_001033865	Rps27a	14033

nex-mRNA in CM of lung culture with TCM. A list of top 20 RNA in lung CM, which was stimulated by TCM, including *βactin* and *IL1β*. (Microarray data were deposited in GSE161219.)

SupplementaryTable 3

Gene symbol	Gene Description	Wt signal	Homo signal	Fold change	mRNA Accession
Igkv6-32	immunoglobulin kappa variable 6-32	975.974741	6605.170101	6.767767467	ENSMUST00000103377
Ighv5-17	immunoglobulin heavy variable 5-17	1067.669936	4406.477124	4.127190412	ENSMUST00000103459
Igkv11-125	immunoglobulin kappa variable 11-125	415.294225	1356.365238	3.266034431	ENSMUST00000103311
Igkv5-48	immunoglobulin kappa variable 5-48	1415.85715	4314.207943	3.047064417	ENSMUST00000103364
Igkv8-19	immunoglobulin kappa variable 8-19	876.834042	2380.105816	2.714431355	ENSMUST00000103389
Retnlg	resistin like gamma	338.4549484	844.3632628	2.494758214	NM_181596
Il1b	interleukin 1 beta	594.4451965	1445.346817	2.431421476	NM_008361
Ighv1-67	immunoglobulin heavy variable V1-67	688.7182039	1665.996899	2.418981943	ENSMUST00000103538
Mmp8	matrix metallopeptidase 8	111.8785771	242.8362518	2.170533967	NM_008611
Igkv14-111	immunoglobulin kappa variable 14-111	1369.591559	2914.211763	2.127796235	ENSMUST00000103320
LOC238440	Ig heavy chain V region	998.7432241	2002.43542	2.004955199	ENSMUST00000103529

Zc3h12a	zinc finger CCCH type containing 12A	247.9834447	232.5585224	0.93779858	NM_153159
Zc3h12b	zinc finger CCCH-type containing 12B	43.30825084	43.92681522	1.01428283	NM_001034907
Zc3h12c	zinc finger CCCH type containing 12C	222.2839543	242.7790293	1.092202224	NM_001162921
Zc3h12d	zinc finger CCCH type containing 12D	692.4710751	142.0219391	0.205094399	NM_172785

List of the candidate genes that might be regulated by Zc3h12d. Gene expression level that was upregulated in *Zc3h12d*-/- mouse spleen compared to that of wild-type shown as fold changes. IL-1β is marked red (top). Zc3h12 family genes in this array data are shown (bottom). (Microarray data were deposited in GSE104002.) Note that *βactin* did not change between wild-type and *Zc3h12d*-/- mice.

SupplementaryTable 4

Gene symbol	Gene Description	Wt signal	Homo signal	Z-score	Fold change	mRNA Accession
Dusp1	dual specificity phosphatase 1	441.6653981	754.3132721	4.774505525	1.707884012	NM_013642
Errfi1	ERBB receptor feedback inhibitor 1	207.7234094	346.1635474	4.566267614	1.666463825	NM_133753
S100a8	S100 calcium binding protein A8	985.0751638	1635.855952	3.577577454	1.660640743	NM_013650
S100a9	S100 calcium binding protein A9	1440.416185	2384.944536	3.557300138	1.655732948	NM_001281852
Il1rn	interleukin 1 receptor antagonist	92.51978447	151.6939776	4.031669422	1.639584209	NM_001039701
Nlrp12	NLR family, pyrin domain containing 12	62.59128977	97.20307171	4.061129896	1.552980807	NM_001033431
Cebpd	CCAAT/enhancer binding protein (C/EBP), delta	112.6989294	171.1550441	3.390912832	1.518692724	NM_007679

List of the genes that were candidate with upregulation in cell nucleus derived from TCM-stimulated mouse spleen. Those were known as nucleus components classified by GO. The fold changes of the gene expression level of *Zc3h12d*-/- mice dividing by that of wild-type. (Microarray data were deposited in GSE104002.)

Supplementary Table 5

Quantitative PCR primers

name	RefSeq accession	Sequence
Mouse CCAAT/enhancer binding protein delta (Cebpd)	NM_007679	Taqman probe: Mm00786711_s1
Human interleukin 1 beta (IL1b)	NM_000576	Taqman probe: Hs01555410_m1
Human ACTB	NM_001101	Taqman probe: Hs01060665_g1
Human RPS27	NM_001030	Taqman probe: Hs01378332_g1
Human RPLP2	NM_001004	Taqman probe: Hs01115128_gH
Human RPS8	NM_001012	Taqman probe: Hs01374307_g1
Mouse IL1b	NM_008361	Taqman probe: Mm00434228_m1
Mouse Actb	NM_007393	Taqman probe: Mm00607939_s1
Mouse Zc3h12d	NM_172785	Taqman probe: Mm01191870_m1
Human IL1b	NM_000576	Forward: CCCTAAACAGATGAAGTGCTCC<br>Reverse: ATCTTCCTCAGCTTGTCCATG<br>Probe: AATCTCCGACCACCACTACAGCAAG
Mouse Dusp 1	NM_013642	Forward: TGTGCCTGACAGTGCAGAAT<br>Reverse: CCTTCCGAGAAGCGTGATAG
Mouse Errfi 1	NM_133753	Forward: GATGCTCGGGCCCCTAAG<br>Reverse: CAAATTTGTAAAGCCCAGGTG
Mouse Nlrp12	NM_001033431	Forward: TCCAGACTCAGTCCACATACT<br>Reverse: GATCAGGTTGGAGTTGGTACAG
Mouse S100A8	NM_013650	Forward: CCGTCTTCAAGACATCGTTTGA<br>Reverse: GTAGAGGGCATGGTGATTTCCT
Mouse S100A9	NM_009114	Forward: GTCCAGGTCCTCCATGATGT<br>Reverse: GAAGGAAGGACACCCTGACA
HOX transcript antisense (Hotair)	NR_047528	Forward: GCTAAGTCCTTCCAGAGAGAAAG<br>Reverse: GCTCTTACTCTCTCTGCCTTTAC
gene-specific primers for mouse IL1b	NM_008361	mIL1b-621R: CCCAAGGCCACAGGTATTT<br>mIL1b-921R: TTAGAAACAGTCCAGCCCATAC<br>mIL1b-1195R: GTTGACAGCTAGGTTCTGTTCT
Mouse Mmp8	NM_008611	Forward 5’-CCAGCACCTATTCACTACCTC-3’<br>Reverse 5’-AGCATCAAATCTCAGGTGGG-3’<br>Probe 5’-ACCTTCAGACAACCCCATCCAACC-3’
Moues Retnlg	NM_181596	Forward 5’-TGTCCCTCCACTGTAACAAAG-3’<br>Reverse 5’-GGCAAGTATTTCCATCCCGG-3’<br>Probe 5’-CCAAGATCCACAGCCATAGCCACA-3’
Mouse Ighv5-17		Forward 5’-GAGTGGCAAGGAGTTCAAATG-3’<br>Reverse 5’-TTTCCTGGACAACTGCTCTG-3’<br>Probe 5’-TCTCGATGGGTGATGGGAGGTCT-3’
Mouse Gapdh	NM_008084	Forward 5’-CTTTGTCAAGCTCATTTCCTGG-3’<br>Reverse 5’-TCTTGCTCAGTGTCCTTGC-3’<br>Probe 5’-CACCCTGTTGCTGTAGCCGTATTCA-3’

List of primers used for qPCR
