# Supplementary material for: Extracellular mRNA transported to the nucleus exerts translation-independent function
Source: Nat Commun. 2021 Jun 16;12:3655. doi: 10.1038/s41467-021-23969-1 (PMC8208975; doi:10.1038/s41467-021-23969-1)
Supplement: Supplementary file 2 — Reporting Summary [file 41467_2021_23969_MOESM2_ESM.pdf]

nature research

Corresponding author(s): Sachie Hiratsuka

Last updated by author(s): Apr 16, 2021

Reporting Summary

Nature Research wishes to improve the reproducibility of the work that we publish. This form provides structure for consistency and transparency in reporting. For further information on Nature Research policies, see our Editorial Policies and the Editorial Policy Checklist.

Statistics

For all statistical analyses, confirm that the following items are present in the figure legend, table legend, main text, or Methods section.

n/a	Confirmed	
☐	☒	The exact sample size (n) for each experimental group/condition, given as a discrete number and unit of measurement
☐	☒	A statement on whether measurements were taken from distinct samples or whether the same sample was measured repeatedly
☐	☒	The statistical test(s) used AND whether they are one- or two-sided<br>*Only common tests should be described solely by name; describe more complex techniques in the Methods section.*
☒	☐	A description of all covariates tested
☐	☒	A description of any assumptions or corrections, such as tests of normality and adjustment for multiple comparisons
☐	☒	A full description of the statistical parameters including central tendency (e.g. means) or other basic estimates (e.g. regression coefficient) AND variation (e.g. standard deviation) or associated estimates of uncertainty (e.g. confidence intervals)
☐	☒	For null hypothesis testing, the test statistic (e.g. F, t, r) with confidence intervals, effect sizes, degrees of freedom and P value noted<br>*Give P values as exact values whenever suitable.*
☒	☐	For Bayesian analysis, information on the choice of priors and Markov chain Monte Carlo settings
☒	☐	For hierarchical and complex designs, identification of the appropriate level for tests and full reporting of outcomes
☐	☒	Estimates of effect sizes (e.g. Cohen's d, Pearson's r), indicating how they were calculated

Our web collection on statistics for biologists contains articles on many of the points above.

Software and code

Policy information about availability of computer code

Data collection	FACSAria III (BD Bioscience), FACSCelesta (BD Bioscience), MoFlo Astrios (Beckman Coulter), Cytomics FC500 (Beckman Coulter), LSM-710 (Carl Zeiss), TCS SP8 (Leica), SPC-130-EM (Becker & Hickl), StepOnePlus (Thermo)
Data analysis	FlowJo v7.6 (FlowJo, LLC), FACSDiva v8 (BD Bioscience), Leica Application Suite X v3 (Leica), Igor Pro v7 (Wavemetrics), ImageJ v1.52 (NIH) , Prism v8 (Graphpad)

For manuscripts utilizing custom algorithms or software that are central to the research but not yet described in published literature, software must be made available to editors and reviewers. We strongly encourage code deposition in a community repository (e.g. GitHub). See the Nature Research guidelines for submitting code & software for further information.

Data

Policy information about availability of data

All manuscripts must include a data availability statement. This statement should provide the following information, where applicable:

- Accession codes, unique identifiers, or web links for publicly available datasets
- A list of figures that have associated raw data
- A description of any restrictions on data availability

No restriction on data availability. Microarray data (GSE76235, GSE161219, GSE104002) are deposited in the Gene Expression Omnibus (GEO) database (https://www.ncbi.nlm.nih.gov/geo). Other data are available from the corresponding author upon request. Source data are provided with this paper.

Field-specific reporting

Please select the one below that is the best fit for your research. If you are not sure, read the appropriate sections before making your selection.

☒ Life sciences ☐ Behavioural & social sciences ☐ Ecological, evolutionary & environmental sciences

For a reference copy of the document with all sections, see nature.com/documents/nr-reporting-summary-flat.pdf

Life sciences study design

All studies must disclose on these points even when the disclosure is negative.

Sample size	Sample size was not predetermined based on statistical methods, but were used chosen based on the previous study in which similar experiments were conducted (Hiratsuka, et al. EMBO Mol Med (2018) e8643). The numbers of independent experiments are indicated in the figure legends.
Data exclusions	No data were excluded from analyses in the experiments.
Replication	At least two independent biological replications for all assays, and noted in the figure legend. All biological replications confirmed the similar results.
Randomization	For the xenograft model, animals were randomly assigned into groups receiving tumor cell injection. In other mouse experiments, mice for a given genotype was randomized. All the cell culture wells were randomized into control and test groups.
Blinding	Blinding is not required as all data was collected by objective measurements.

Reporting for specific materials, systems and methods

We require information from authors about some types of materials, experimental systems and methods used in many studies. Here, indicate whether each material, system or method listed is relevant to your study. If you are not sure if a list item applies to your research, read the appropriate section before selecting a response.

Materials & experimental systems

n/a	Involved in the study	
☐	☒	Antibodies
☐	☒	Eukaryotic cell lines
☒	☐	Palaeontology and archaeology
☐	☒	Animals and other organisms
☐	☒	Human research participants
☒	☐	Clinical data
☒	☐	Dual use research of concern

Methods

n/a	Involved in the study	
☒	☐	ChIP-seq
☐	☒	Flow cytometry
☒	☐	MRI-based neuroimaging

Antibodies

Antibodies used	Anti-ZC3H12D antibodies for mouse (Abcam ab1000862, dilution 1:100-200), E14 (Santa Cruz Biotechnology, sc-169839, dilution 1:100-200) and M15 (Santa Cruz Biotechnology, sc-169840, dilution 1:100) for FACS analysis, and Anti-ZC3H12D (Proteintech, 24991-1-AP, dilution 1:100) for Western blotting. Anti-ZC3H12D antibodies for human (Abcam, ab1000862, dilution 1:100), anti-ZC3H12A antibody for human (N3C3, GeneTex, GTX110807, dilution 1:100), and histone H2AXS139ph (phosphor Ser139) antibody (GT2311, GeneTex, GTX628789, dilution 1:200) were used in IHC analyses. For mouse cell sorting and FACS analyses, the following were used. For mouse CD11c: PE anti-mouse CD11c (BioLegend, 117308 dilution 1:100-200); isotype control, PE Armenian Hamster IgG (eBioscience, 12-4888-81, dilution 1:100); Alexa Fluor 647 anti-mouse CD11c (BioLegend, 11732, dilution 1:100); and isotype control, Alexa Fluor 647 Armenian Hamster IgG (BioLegend, 400526, dilution 1:100). For mouse NK1.1: Brilliant Violet 421 anti-mouse NK-1.1 (BioLegend, 108741, dilution 1:100); isotype control, Brilliant Violet 421 mouse IgG2a, κ (BioLegend, 400260, dilution 1:100); APC anti-mouse NK-1.1 Antibody (BioLegend, 108710, dilution 1:100); and isotype control, APC mouse IgG2a, κ (BD Pharmingen, 550882, dilution 1:100). For B220: PE/Cy7 anti-mouse/human CD45R/B220 Antibody (BioLegend, 103222, dilution 1:200); isotype control, PE/Cy7 Rat IgG2a, κ (BioLegend, 400522, dilution 1:200); APC anti-mouse/human CD45R/B220 Antibody (BioLegend, 103212, dilution 1:200); and isotype control, APC Rat IgG2a, κ (BD Pharmingen, 554690, dilution 1:200). For CD45: PE/Cy7 anti-mouse CD45 Antibody (BioLegend, 304014, dilution 1:200) and isotype control, PE/Cy7 Rat IgG2b (BioLegend, 400127, dilution 1:200). In cell sorting for human CD56: PE anti-human CD56 (NCAM) Antibody (BioLegend, 362524, dilution 1:50-100) and isotype control, Mouse IgG1, κ (BioLegend, 400111, dilution 1:50-100). In cell sorting for human CD3: Alexa Fluor 488 anti-human CD3 antibody (BioLegend, 300454, dilution 1:100) and isotype control, Mouse IgG1, κ (BioLegend, 400132, dilution 1:100). Anti-IFNγ antibodies for mice (D-17, Santa Cruz Biotechnology, sc-9344, dilution 1:200) and humans (B27, BioLegend, 506501, dilution 1:200) were used in cell staining. Human IL2 (BioLegend, 791902) and human IL12 (Proteintech, HZ-1256) were used in the cell culture. Anti-SC35 (Abcam, ab204916, dilution 1:100), anti-SFPQ (C23, MBL, RN014MW, dilution 1:1000), anti-PML (MBL, K0196-3, dilution 1:1000), anti-fibrillarin (38F3, Biolegend, 905001, dilution 1:500), and Alexa Fluor 647 anti-NKG2D (Biolegend, 320825, dilution 1:100) were also used in cell

	staining. Anti-DNA polymerase II (F-12, Santa Cruz Biotechnology, sc-55492, dilution 1:100) was used for immunoprecipitation. Anti-DDDDK was used to detect FLAG-tag in western blotting.
Validation	Anti-ZC3H12D antibodies for mouse (Abcam, ab1000862), validated by flow cytometry. Detailed data is presented in the manuscript (Figure S10);<br>Anti-ZC3H12D (Proteintech, 24991-1-AP, dilution 1:100), validated by Western blotting;<br>Anti-ZC3H12D antibodies for human (Abcam, ab1000862), validated by Western blotting;<br>Anti-ZC3H12A antibody for human (N3C3, GeneTex, GTX110807), validated by immunofluorescence;<br>Anti-histone H2AXS139ph (phosphor Ser139) antibody (GT2311, GeneTex, GTX628789), validated by immunofluorescence;<br>PE anti-mouse CD11c (BioLegend, 117308), validated by flow cytometry;<br>Alexa Fluor 647 anti-mouse CD11c (BioLegend, 11732), validated by flow cytometry;<br>Brilliant Violet 421 anti-mouse NK-1.1 (BioLegend, 108741), validated by flow cytometry;<br>APC anti-mouse NK-1.1 Antibody (BioLegend, 108710), validated by flow cytometry;<br>PE/Cy7 anti-mouse/human CD45R/B220 Antibody (BioLegend, 103222), validated by flow cytometry;<br>APC anti-mouse/human CD45R/B220 Antibody (BioLegend, 103212), validated by flow cytometry;<br>PE/Cy7 anti-mouse CD45 Antibody (BioLegend, 304014), validated by flow cytometry;<br>PE anti-human CD56 (NCAM) Antibody (BioLegend, 362524), validated by flow cytometry;<br>Alexa Fluor 488 anti-human CD3 antibody (BioLegend, 300454), validated by flow cytometry;<br>Anti-IFNγ antibodies for mice (D-17, Santa Cruz Biotechnology, sc-9344), validated by immunofluorescence;<br>Anti-IFNγ antibodies for humans (B27, BioLegend, 506501), validated by flow cytometry;<br>Anti-SC35 (Abcam, ab204916), validated by immunofluorescence;<br>anti-SFPQ (C23, MBL, RN014MW), validated by immunofluorescence;<br>anti-PML (MBL, K0196-3), validated by immunofluorescence;<br>anti-fibrillarin (38F3, Biolegend, 905001), validated by immunofluorescence;<br>Alexa Fluor 647 anti-NKG2D (Biolegend, 320825), validated by flow cytometry;<br>Anti-DNA polymerase II (F-12, Santa Cruz Biotechnology, sc-55492), validated by Western blotting and immunofluorescence;<br>Anti DDDDK (MBL, PM020), validated by Western blotting;

Eukaryotic cell lines

Policy information about cell lines

Cell line source(s)	E0771 (ATCC, CRL-3461) breast cancer cells were originally established by Dr Sirotnak (Memorial Sloan–Kettering Cancer Center, New York, NY) and provided by Dr Mihich (Roswell Park Memorial Institute, Buffalo, NY). Lewis lung carcinoma (RCB0558), RAW264.7 (RCB0535), THP-1 (RCB1189), 293T (RCB2202) and B16 melanoma cells (RCB1283) were purchased from Riken BRC. LLC (3LL) cells (JCRB Cell Bank, JCRB1348) and the 3LL in vivo passage line were supplied by the Japanese Foundation for Cancer Research (Tokyo). MCF7(HTB-22), MDAMB-231 (HTB-26), T47D (HTB-133), and SKBR3 (HTB-30), were obtained from ATCC. HCC1954 cells (CRL-2338) and 786-O cells (CRL-1932) were purchased from ATCC.
Authentication	Cells were not authenticated.
Mycoplasma contamination	The cell lines were tested for negative mycoplasma contamination by using MycoAlert mycoplasma detection kit (Lonza, LT-07-218).
Commonly misidentified lines<br>(See ICLAC register)	No commonly misidentified cell lines were used in this study.

Animals and other organisms

Policy information about studies involving animals; ARRIVE guidelines recommended for reporting animal research

Laboratory animals	Mice, C57BL6, male and female, 7-15 weeks. The following strains were used: Zc3h12d-/-, Zc3h12d+/+.
Wild animals	No wild animals were involved in this study.
Field-collected samples	This study did not involve animals collected from the field.
Ethics oversight	A study protocol was approved by the Animal Care and Use Committee of Shinshu University and Tokyo Women's Medical University.

Note that full information on the approval of the study protocol must also be provided in the manuscript.

Human research participants

Policy information about studies involving human research participants

Population characteristics	Primary human cells were purchased from Applied Cell Biology Research Institute (ACBRI 468) and Lonza (CC-2702).
Recruitment	The cells were commercially available.
Ethics oversight	Cells were received from full consent donors with IRB-approved protocols.

Note that full information on the approval of the study protocol must also be provided in the manuscript.

Flow Cytometry

Plots

Confirm that:

- [x] The axis labels state the marker and fluorochrome used (e.g. CD4-FITC).
- [x] The axis scales are clearly visible. Include numbers along axes only for bottom left plot of group (a 'group' is an analysis of identical markers).
- [x] All plots are contour plots with outliers or pseudocolor plots.
- [x] A numerical value for number of cells or percentage (with statistics) is provided.

Methodology

Sample preparation	Human peripheral blood monocytes (PBMCs) and mouse spleen cells were suspended in PBS with 1% BSA.
Instrument	FACSAria III (BD Bioscience), FACSCelesta (BD Bioscience), MoFlo Astrios (Beckman Coulter), Cytomics FC500 (Beckman Coulter)
Software	FlowJo v7.6 (FlowJo, LLC), FACSDiva v8 (BD Bioscience)
Cell population abundance	Forward and side scatter gating isolated cell population. The purity was approximately 70% of the starting material. The total number of cells counted was more than 10,000. PBMCs were further analyzed by using Alexa488-anti-CD3, PE-anti-CD56, and anti-ZC3H12D to find out that 13.2% of gated cells were ZC3H12D positive. Analysis of mouse spleen cells revealed that a few percent cells were B220+CD11c+NK1.1+.
Gating strategy	Cells were gated using the forward versus side scatter log area plot. Gating strategy is provided in Figure S10.

- [x] Tick this box to confirm that a figure exemplifying the gating strategy is provided in the Supplementary Information.
