# Supplementary figures and images for: Extracellular mRNA transported to the nucleus exerts translation-independent function
Source: Nat Commun. 2021 Jun 16;12:3655. doi: 10.1038/s41467-021-23969-1 (PMC8208975; doi:10.1038/s41467-021-23969-1)

GeL-data related to Fig. 2b

ZC3H12D-overexpressed RAW (ZC+RAW) cells  
(Full gel data-Western blot)

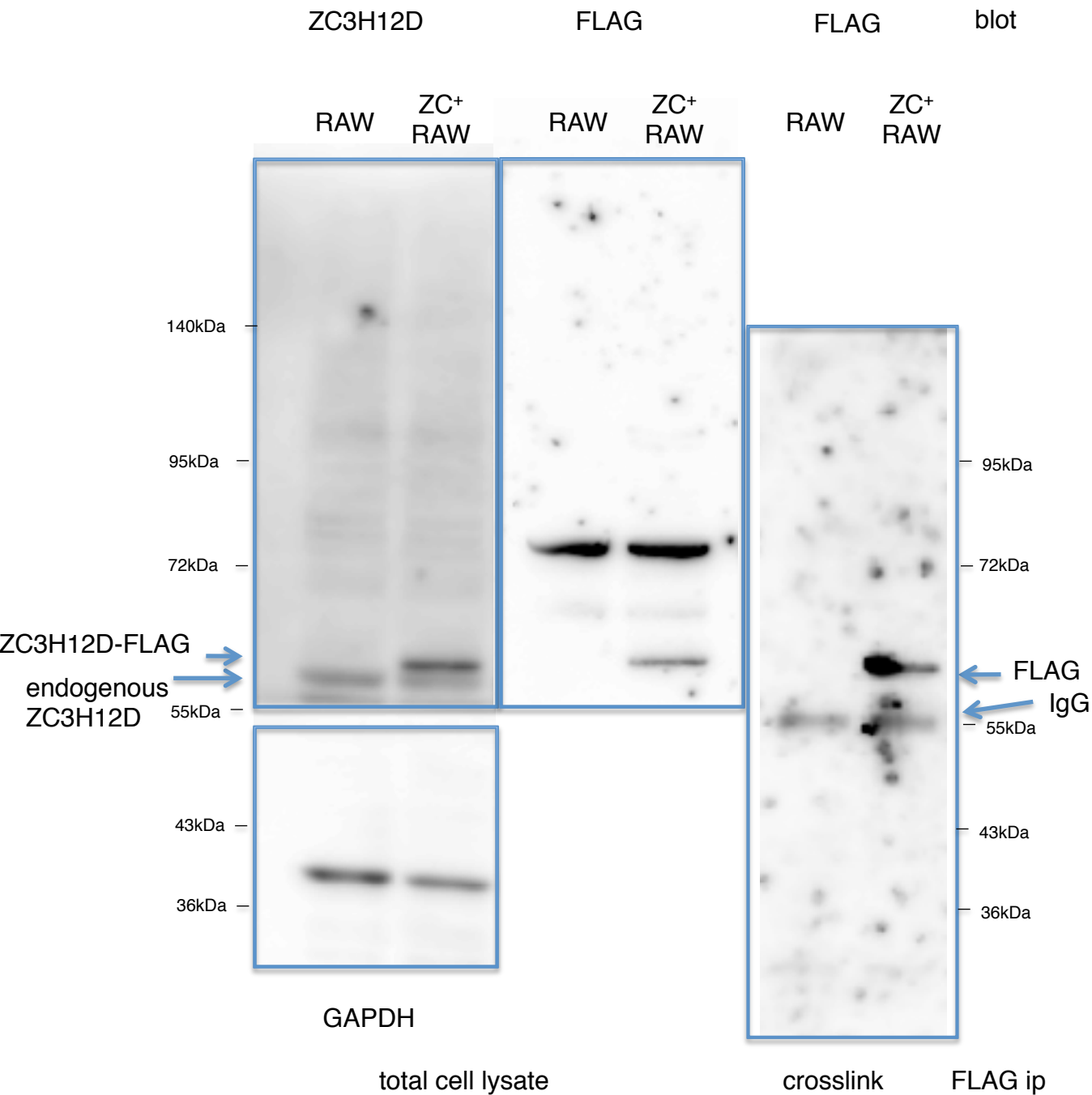

Supplement: Supplementary file 4 — Source Data [file 41467_2021_23969_MOESM4_ESM.zip › 5-SouceData-2/7-GeL-Data.pdf]
